# AdcAII of *Streptococcus pneumoniae* Affects Pneumococcal Invasiveness

**DOI:** 10.1371/journal.pone.0146785

**Published:** 2016-01-11

**Authors:** Lindsey R. Brown, Steven M. Gunnell, Adam N. Cassella, Lance E. Keller, Lisa A. Scherkenbach, Beth Mann, Matthew W. Brown, Rebecca Hill, Nicholas C. Fitzkee, Jason W. Rosch, Elaine I. Tuomanen, Justin A. Thornton

**Affiliations:** 1 Department of Biological Sciences, Mississippi State University, Mississippi State, MS, 39762, United States of America; 2 Department of Chemistry, Mississippi State University, Mississippi State, MS, 39762, United States of America; 3 Department of Microbiology, University of Mississippi Medical Center, Jackson, MS, 39216, United States of America; 4 Department of Infectious Diseases, St. Jude Children’s Research Hospital, Memphis, TN, 38105, United States of America; Instituto Butantan, BRAZIL

## Abstract

Across bacterial species, metal binding proteins can serve functions in pathogenesis in addition to regulating metal homeostasis. We have compared and contrasted the activities of zinc (Zn^2+^)-binding lipoproteins AdcA and AdcAII in the *Streptococcus pneumoniae* TIGR4 background. Exposure to Zn^2+^-limiting conditions resulted in delayed growth in a strain lacking AdcAII (ΔAdcAII) when compared to wild type bacteria or a mutant lacking AdcA (ΔAdcA). AdcAII failed to interact with the extracellular matrix protein laminin despite homology to laminin-binding proteins of related streptococci. Deletion of AdcA or AdcAII led to significantly increased invasion of A549 human lung epithelial cells and a trend toward increased invasion *in vivo*. Loss of AdcAII, but not AdcA, was shown to negatively impact early colonization of the nasopharynx. Our findings suggest that expression of AdcAII affects invasiveness of *S*. *pneumoniae* in response to available Zn^2+^ concentrations.

## Introduction

*Streptococcus pneumoniae* normally colonizes the human nasopharynx asymptomatically, but can also cause serious infections, including pneumonia, septicemia, and bacterial meningitis. Thus, the pneumococcus is able to adapt to a wide variety of host environments. An important variable in these diverse niches is the concentration of metals, such as Zn^2+^. Limitation of Zn^2+^ results in growth arrest and thus Zn^2+^ transport is critical to bacterial multiplication and, indirectly, to progression of disease. A key question is whether these metal transporters have additional functions such as directly affecting adherence to host cells or the extracellular matrix.

The LraI (lipoprotein receptor antigen I) family of lipoproteins represents one group of metal transporters. Previously characterized proteins within the LraI family, including the manganese-binding protein PsaA from *S*. *pneumoniae*, play roles in both adhesion and metal transport [1, 2]. Adhesion of pneumococci to nasopharyngeal epithelial cells is reduced by antibodies to PsaA, [3] and PsaA has been proven essential for virulence in animal models when inoculated intranasally [4].

AdcAII is homologous to members of the LraI family, several of which have been characterized in other gram-positive pathogenic species. Lmb expressed by *Streptococcus agalactiae* and Lsp or Lbp expressed by *Streptococcus pyogenes* are both known to confer laminin-binding properties that promote adhesion and invasion. In *S*. *agalactiae*, a mutant lacking Lmb shows decreased adherence to human laminin [5] and reduced invasion into human brain microvascular endothelial cells (HBMEC) [6]. *S*. *pyogenes* mutants lacking Lsp are defective in adhesion and invasion of epithelial cells *in vitro* and are also highly attenuated in a murine subcutaneous ulcer model [7, 8]. This raises the question of what role AdcAII plays in pneumococcal physiology.

Biophysical and structural studies have demonstrated that AdcAII of *S*. *pneumoniae* serves as a metal-binding receptor (MBR) for Zn^2+^, given the presence of a flexible binding loop commonly seen in other Zn^2+^-binding transporters [9]. Additional studies identified that AdcAII actually serves as a second Zn^2+^ transporter, along with AdcA of the AdcCBA operon [10], regulating intracellular levels of Zn^2+^ in pneumococcus [11]. Only recently, have the relative contributions of AdcA and AdcAII been investigated [12]. Crystal structure analysis indicated that both proteins contain a highly homologous Zn^2+^-binding domain but AdcA has a second Zn^2+^-binding domain, absent in AdcAII [13]. Both proteins transfer Zn^2+^ to the AdcBC transporter and have overlapping functions in Zn^2+^ homeostasis. However, it has been suggested that AdcAII may play a role in other areas of pathogenesis since histidine triad proteins, which are known to be involved in adherence, are encoded directly downstream [14, 15]. AdcAII and pneumococcal histidine triad proteins are upregulated during invasive pneumococcal disease, [16] and expression of *adcAII* and *pht* genes is affected by availability of Zn^2+^ [17, 18]. Additionally, transposon mutagenesis identified AdcAII, AdcB, and AdcC as being involved in colonization [19]. Fluctuations in available Zn^2+^ concentrations within a host could therefore serve to alter expression of these surface proteins, which in turn could have effects on the pathogen’s virulence.

Given the clinical significance of pneumococcal infections and previous work showing the importance of AdcAII homologs to virulence in other species, we investigated the relative contribution of AdcA and AdcAII to processes including adhesion and invasion. Here we show that AdcA and AdcAII mutants display distinctly different phenotypes during colonization and during growth in zinc-limiting environments.

## Materials and Methods

### Bacterial strains and growth conditions

Novablue strain *E*. *coli* (Merck KGaA, Darmstadt, Germany) was grown at 37°C in Luria-Bertani (LB) broth with agitation or on LB agar supplemented, when appropriate, with spectinomycin (50μg/mL). *S*. *pneumoniae* serotype 4 strains TIGR4 and the unencapsulated TIGR4 derivative T4R were grown on tryptic soy agar plates supplemented with 5% defibrinated sheep blood, Kaighn’s medium (F-12K; ATCC 3–2004) with 2mM L-glutamine and 1500mg/L sodium bicarbonate supplemented with 10% FBS, or in Todd-Hewitt broth (BD Biosciences, Sparks MD) supplemented with 0.5% yeast extract [20]. Isogenic mutants of T4R lacking AdcA (ΔAdcA), and AdcAII (ΔAdcAII) were created using splicing by overlap extension (SOE) PCR method using erythromycin and spectinomycin antibiotic cassettes [21] and standard *S*. *pneumoniae* transformation procedures. Mutants lacking AdcAII were isolated by selection on blood agar plates supplemented with erythromycin (0.5μg/mL) and ΔAdcA mutants were isolated by selection on blood agar plates supplemented with spectinomycin (500μg/mL). Double mutants (ΔAdcA/ΔAdcAII) were selected for using blood agar plates supplemented with both erythromycin and spectinomycin. All mutants were confirmed by PCR. Primer sequences used for construction of mutants are listed in S1 Table. Complemented mutants of AdcAII (ΔAdcAII+) were created by cloning the *adcAII* gene from T4R by amplifying the gene by PCR followed by digestion of the product with EcoRI and XmaI restriction enzymes (New England Biolabs). Primers were LMB3 (CGCGATCCCGGGGGTTAAGGAGTTGTTCATGAAGAAAC) and LMB4 (CCGCGGAATTCCTTTCCTCACTTTAATTCTTCTGC) with restriction sites underlined. Following enzymatic digestion, the gene was ligated with the pNE-1 pneumococcal shuttle vector that was similarly digested. The ligation was transformed into *E*. *coli* strain DH5α. Purified plasmid was then used to transform ΔAdcAII strains by standard methods and complemented mutants were selected by plating on blood agar plates supplemented with erythromycin (0.5μg/mL) and spectinomycin (500μg/mL). Complementation of AdcAII was confirmed by western blot (S1 Fig).

### DNA Manipulation

Chromosomal DNA from TIGR4 *S*. *pneumoniae* was purified by standard methods. Plasmid DNA (pNE1) was prepared from *E*. *coli* using GenElute HP Plasmid Midiprep kit (Sigma-Aldrich, St. Louis, MO). PCR reactions contained 100-200ng of chromosomal DNA, primers at 200nM, 200μM of each dNTP, indicated buffer, and Takara Taq Ex. After initial incubation at 94°C, reactions completed 30 cycles of incubation at 94°C for 1 minute, 53°C for 1 minute, 72°C for 5 mins, with final incubations at 4°C.

### Quantitative Real-time PCR

*S*. *pneumoniae* T4R was grown in Todd-Hewitt medium supplemented with 0.5% yeast extract to O.D._600_ of 0.6 prior to the addition of Zn^2+^-chelating agent TPEN (30μM) (Sigma-Aldrich P4413) or ZnS0_4_ (100μM). After addition of TPEN or ZnS0_4_, bacteria were incubated at 37°C for 5 mins. At the 5 min time point 2mL of bacterial culture was added to 4mL of RNAprotect (Qiagen). Samples were incubated at room temperature for 5 mins. Two mL of bacterial culture in RNAprotect was pelleted for 5 mins at 13,000rpm, pellet was resuspended in 1mL of cold RNase free PBS, and samples were centrifuged again for 5 mins at 13,000rpm. Supernatant was decanted and pellet was resuspended in 400μL RLT buffer (Qiagen) with 2-Mercaptoethanol (Sigma-Aldrich M6250). Samples were sonicated three times (15 seconds), 600μL of RLT buffer was added to each sample, and sample was transferred to 500μL of 0.7mm Zirconia beads. Samples were bead beat for 5 mins using Mini-BeadBeater 16 (BioSpec Products). Lysate was centrifuged on tabletop centrifuge for 1 min. 700μL of sample was placed over Qiashredder (Qiagen) per manufacturer’s instructions. 100% Ethanol was added at 0.6 volume of the sample. RNA was then purified using a Qiagen RNeasy Mini Kit (Qiagen), optional on-column DNase treatment was performed, RNA was quantitated using a Qubit, and 50ng from each sample was used to synthesize cDNA using a Maxima First Strand cDNA synthesis kit (Thermo Scientific). cDNA products were diluted 1:100 and 1μL of cDNA was used as template for real-time PCR using (Luminaris Color HiGreen High ROX qPCR Master Mix (Thermo Scientific) per manufacturer’s instructions. Oligonucleotide sequences for *adcAII* were 5’ TCGGATGATTCAGTCAAGTAGTG and 5’ CAGACTTCCTGCCCAAGATT. Oligonucleotide sequences for *adcA* were 5’ GATAAGGCTTACGCAGAAGGT and 5’ GTCCATAGTCCAAGGCAAGATAG. Gyrase A served as a control gene and oligonucleotide sequences were 5’ ATGACCTCTTGGCTCTGATTG and 5’ CAACTCTGTACGGCGCTTAT. Relative fold changes were determined by ΔΔCt analysis comparing samples treated with TPEN or ZnSO_4_ to untreated controls, using GyrA as an internal housekeeping control gene.

### Adhesion assay and invasion assay

Adhesion and invasion assays were performed in the A549 cell line. Cells were seeded to 80–90% confluence in a 24-well plate (Invitrogen). Cells were incubated with 1 x 10^6^ CFU/mL bacteria at 37°C in 5% CO_2_. After 30 mins, cells were washed with D-PBS with Calcium and Magnesium (D-PBS +Ca/Mg) three times, trypsinized, and diluted 1:10 in PBS. 10μL was plated on blood agar and incubated at 37°C overnight. For invasion assays, cells were prepared in the same manner as for the adhesion assays. Cells were incubated with 1 x 10^6^ CFU/mL bacteria for 2 hrs. Cells were then washed three times with D-PBS +Ca/Mg, and incubated with appropriate cell culture media with penicillin/gentamycin (10μg/mL and 250μg/mL, respectively) for 1 hr. Cells were again washed with D-PBS +Ca/Mg, trypsinized, and lysed with Triton X-100 (0.0125% final conc.). Serial dilutions were plated on TSA supplemented with 5% sheep blood and incubated overnight at 37°C in 5% CO_2_. Some experiments were performed in the same manner with the exception that bacteria were inoculated into media containing ZnS0_4_ (200μM) prior to being seeded onto A549 monolayers. Each experimental condition was performed in three replicate wells and was repeated at least three times.

### Fluorescence Microscopy

Invasion assays were performed as described above with the exception that A549 cells were seeded to 80% confluency in 2 well chamber slides (Nalge Nunc Int, Rochester, NY). After 2 hrs of contact with bacteria followed by 1 hr in penicillin/gentamycin supplemented media, cells were washed with D-PBS +Ca/Mg and fixed with 4% paraformaldehyde (in PBS) at 25°C for 10 mins. Cells were then rinsed three times with PBS and blocked with PBS plus 0.5% BSA, 4% mouse serum, and 0.5% Triton-X 100 for 15–30 mins. Cells were incubated with anti-TEPC-15 primary antibody (Sigma-Aldrich M-1421, specific for phosphorylcholine at 1:40) overnight at 4°C, rinsed two times with PBS for 5 mins each, prior to being incubated with goat anti-mouse IgA conjugated to rhodamine (Southern Biotech 1040–03 Birmingham, AL, at 1:100) for 1 hr. Cells were then rinsed two times with PBS for 5 mins each then 0.5mL of 300nM DAPI was added and incubated for 20 mins at room temperature. Cells were then rinsed three times with PBS before the chambers were removed, then 10μL of VECTASHIELD Mounting Medium (Vector H-1000) was added, and a cover slip was applied. Cells were visualized by fluorescence microscopy using a Zeiss Axioskop 2 Plus.

### Mouse challenges

All experiments involving animals were planned and conducted in accordance with guidelines of the MSU Institutional Animal Care and Use Committee. The committee specifically approved procedures performed in the study presented here. C57BL/6 mice were maintained at Biosafety Level 1 and 2 facilities in the Mississippi State University Animal Facility. Six-week old female mice were anesthetized with isoflurane and challenged intranasally with either 20μL of PBS containing 7x10^3^ (for colonization) or 100μl of PBS containing 3.6x10^4^ (for invasion) of TIGR4, ΔAdcA, or ΔAdcAII. At 24 hrs and 48 hrs post-infection, mice were humanely euthanized by deep isoflurane inhalation followed by cervical dislocation and confirmed by incubation for 5 min under CO_2_. During the time course of the infections, no mice became severely ill or required early/humane endpoint sacrifices. This was determined by monitoring physical or behavioral changes including, weight loss, dehydration, loss of balance, or becoming moribund. Nasal washes, nasal tissues, and blood samples were collected. Bacterial titers were determined by plating serial dilutions on tryptic soy agar plates supplemented with 5% defibrinated sheep blood with neomycin (20μg/mL), followed by a16 hr incubation at 37°C.

### Microarray analysis

To determine if loss of AdcAII has an effect on global gene expression in *S*. *pneumoniae*, microarray analysis was performed to compare transcript levels between T4R and its isogenic mutant ΔAdcAII. T4R and the ΔAdcAII knockout strain were cultured in C+Y media. Bacterial RNA was harvested from mid-log-phase cultures by the Qiagen RNeasy Mini Kit (Qiagen). Microarray experiments were performed as described previously [16]. Whole genome *S*. *pneumoniae* oligonucleotide microarrays obtained from the PFGRC at the J. Craig Venter Institute (http://pfgrc.jcvi.org/) were used for microarray experiments (lot numbers 6QSP11062007A and 6QSP11012007A). The array consisted of 70-mer oligonucleotide probes representing 2060 open reading frames from *S*. *pneumoniae* strain TIGR4 and 457 from strains G54 and R6, and each probe sequence was spotted at least five times on the array. Data have been submitted to NCBI under accession GSM358422, GSM358242, GSM358241, GSM358231, GSM358229 and GSM358101. To analyze the microarray data, a series of filtration algorithms was applied to eliminate spots with poor-quality data. Spots flagged (as bad, absent or not found) by the image analysis software GenePix 6.0 (Axon Corp., Union City, CA) and spots having a signal-to-noise ratio less than 1.5 or a background-corrected signal reading less than 20 in both Cy3and Cy5 channels were excluded from analysis. To correct the intensity bias, loess normalization was applied to each microarray. Of at least five replicate spots on each array, only genes whose corresponding spots passed the filtering criterion at least 50% of the times were reported in further analysis. Of the 11960 spots dedicated to TIGR4, approximately 16.8% were eliminated because of poor quality or low signal intensity. These were typically gene specific, with all five spots failing to give adequate signal intensity, indicative of either poor hybridization to the oligonucleotide probe or low abundance of the specific transcript. For each experiment, results were summarized from three independent microarray replicates, which included one dye flip experiment for adjusting the dye bias. The average of the fold changes from at least two replicated experiments for each gene was calculated, and those with a minimum two-fold change were listed in respective tables. All genes included in the table had a *P*-value less than 0.05, using a *t*-distribution test.

### Western Blotting

Bacterial cultures were grown in Todd-Hewitt medium supplemented with 0.5% yeast extract to an O.D._600_ of 0.8. Cultures were centrifuged and pellets were lysed in 100μL Pneumococcal lysis buffer (0.01% SDS, 0.1% DOC, and 0.015M Na Citrate). Equal amounts of protein were determined using a BCA Protein Assay (Pierce) loaded in Mini-Protean gel (BIO-RAD), transferred using a semi-dry transfer method (BIO-RAD Trans Blot SD), blocked with 5% non-fat milk, and probed with anti-Sp0463 (pilus) as the primary antibody, and goat-anti mouse conjugated to HRP as the secondary antibody. For western blot confirmation of AdcAII complementation, blotted lysates were probed with polyclonal rabbit antiserum against AdcAII (Rockland labs).

### Production of recombinant AdcAII constructs

The AdcAII gene lacking the signal sequence was amplified from TIGR4 chromosomal DNA using primers BMLMBF2 and BMLMBR containing EcoRI and NdeI restriction sites, respectively. Primer sequences listed in S1 Table. Restriction digests included 100-200ng of PCR product or plasmid DNA, enzymes, indicated buffer, and bovine serum albumin (BSA) when indicated by manufacturer (New England Biolabs). Digests were incubated for at least 6 hrs at 37°C and DNA was isolated using QIAquick PCR Purification Kit (Qiagen, Valencia, CA) for gene inserts or QIAquick Gel Extraction kit (Qiagen) for plasmids according to manufacturer’s instructions. Restricted PCR products were ligated into previously digested expression vector pET-28 (Invitrogen) prior to transformation into *E*. *coli* using standard molecular biology techniques. Recombinant histidine-tagged protein was purified using HIS-Select Nickel Affinity Gel (Sigma-Aldrich)

### Dot blot

Interaction between AdcAII and human matrix proteins was determined by dot blot analysis as previously described [8]. Using a BIO-DOT vacuum apparatus (Bio-Rad), 2μg, 5μg, and 10μg of collagen (Sigma-Aldrich), fibronectin, and laminin (Sigma-Aldrich) were transferred in 20μL spots onto PVDF membrane (Millipore, Bedford, MA) previously soaked with 100% methanol and rinsed in transfer buffer (4.5% Tris, 22% glycine, 5% methanol). Anti-AdcAII polyclonal antiserum served as a control for binding to the membrane. The blot was blocked for 30 min in PBST containing 2% BSA (Sigma-Aldrich Inc.) prior to incubation with biotinylated AdcAII protein (10μg/mL) in PBST-2% BSA for 2 hrs at room temperature. AdcAII was biotin labeled using the Biotin Labeling Kit (Roche, Indianapolis, IN) per manufacturer’s instructions. After incubation with biotinylated AdcAII, the blot was washed with PBST and incubated with horseradish peroxidase strepavidin (1:5000) (Vector Laboratories, Burlingame, CA) prior to another wash and development using SuperSignal West Pico Chemiluminescent Substrate (Thermo Scientific, Rockford, IL). For a laminin-binding control, recombinant 37/67kDa laminin receptor protein was used as a probe and developed using anti-LR mAb (1:500) (Abcam, Cambridge, MA) and HRP-conjugated goat anti-mouse secondary (1:10000) (Bio-Rad).

### Biophysical Characterization

Circular dichroism (CD) experiments were performed on an Olis DSM 20 spectropolarimeter. Protein concentrations were estimated using an extinction coefficient at 280 nm of 24,410 M^-1^ cm^-1^ [22]. AdcAII was measured in a 0.1 mm pathlength cell at a concentration of 42 μM at 25°C in 25 mM Tris buffer pH 7.5. Data were collected at intervals of 0.5 nm over the far-UV range of 260 to 190 nm. After data collection, spectra were smoothed using the Savitsky-Golay algorithm over a window size of 25 points [23]. The processed data were analyzed using the CDSSTR program to predict the secondary structure content [24, 25]. To facilitate analysis, a convenient Python program was developed, streamlining data analysis for the CDSSTR core program. This program runs on Mac, Linux, and Windows computers and is available at http://fitzkee.chemistry.msstate.edu/software/. The crystal structure of AdcAII (PDB ID 3CX3, chain A) was used for comparison with CD-based secondary structure predictions [9]. Backbone secondary structure in the crystal structure was assigned using DSSP [26].

Dynamic light scattering (DLS) measurements were collected on a Wyatt DynaPro NanoStar instrument under identical conditions as CD measurements. Samples were filtered using an Anotop 10 syringe filter with a 0.02 μm pore size (GE Healthcare Life Sciences, New Brunswick, NJ). Sample loss during filtration was determined to be minimal (< 10%) based on absorbance measurements. Cumulant and regularization analysis were both applied to the DLS data and agreed to within 2 Å with low polydispersity (< 20%). The native state hydrodynamic radius (*R*_H_) was estimated from the crystal structure (PDB 3CX3) by dividing the largest C_α_-C_α_ distance by two (R_H_ ≈ 30.5 Å).

### Statistics

In instances where values from three or more experiments were compared, we used a two-tailed Student’s *t-*test to test for statistical significance. A *P* value less than 0.05 was considered significant.

## Results

### Relative importance of AdcA and AdcAII during Zn^2+^ limitation

**Fig 1:**
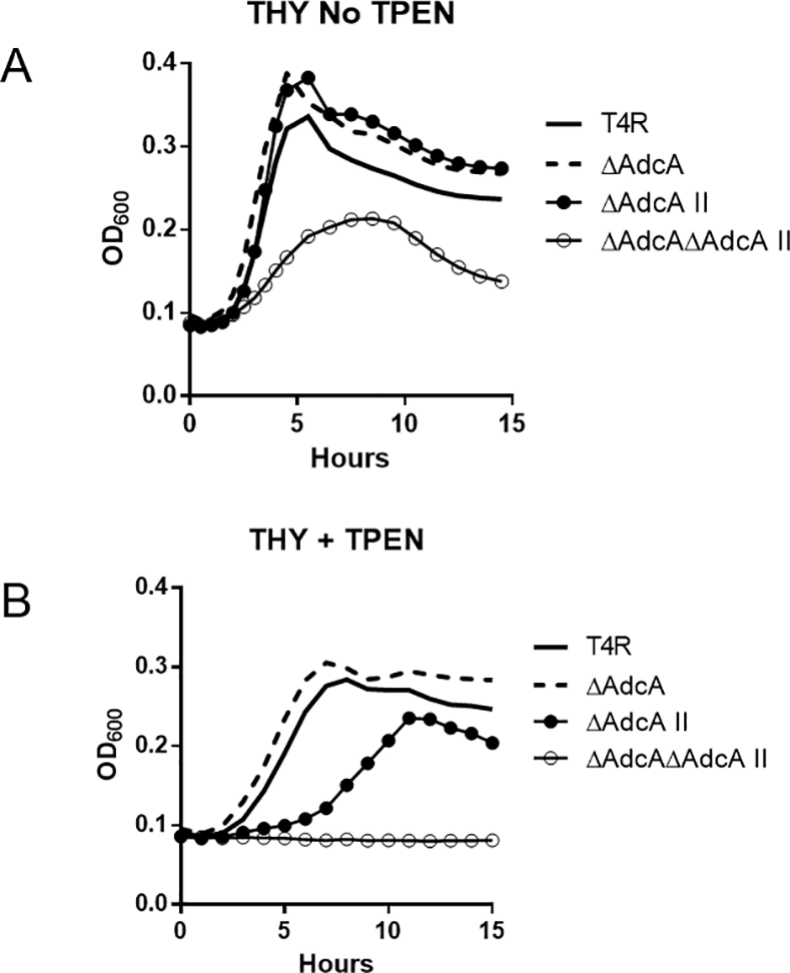
Effects of zinc concentration on growth of Adc mutants. A) Representative growth curve from three runs of T4R wild type and Adc mutants grown in THY media (pH6.5) in the absence (A) or presence (B) of the Zn-specific chelator TPEN (21μM).

Previous work in the laboratory-adapted strains D39 and R6 have shown that expression of both *adcA* and *adcAII* is important for growth in low Zn^2+^ conditions [12, 27]. Using derivatives of the meningitis isolate TIGR4, wild type (T4R) and isogenic mutants ΔAdcA, ΔAdcAII, and ΔAdcA/AdcAII were grown in THY media containing various concentrations of the Zn^2+^-specific chelator, N,N,N',N'-Tetrakis-(2-pyridylmethyl) ethylenediamine (TPEN) [12]. In the absence of TPEN, only the double mutant ΔAdcA/AdcAII displayed a growth defect (Fig 1A). Titration of the TPEN concentration indicated that ΔAdcAII demonstrated delayed growth compared to wild type T4R at concentrations where growth of ΔAdcA was virtually unaffected (Fig 1B). Supplementation of TPEN-containing media with ZnSO_4_ complemented the growth defect of ΔAdcAII, confirming the effect was Zn^2+^-dependent (data not shown). Based on these results, AdcAII appears to be more important for growth in Zn^2+^limiting conditions than AdcA.

**Fig 2:**
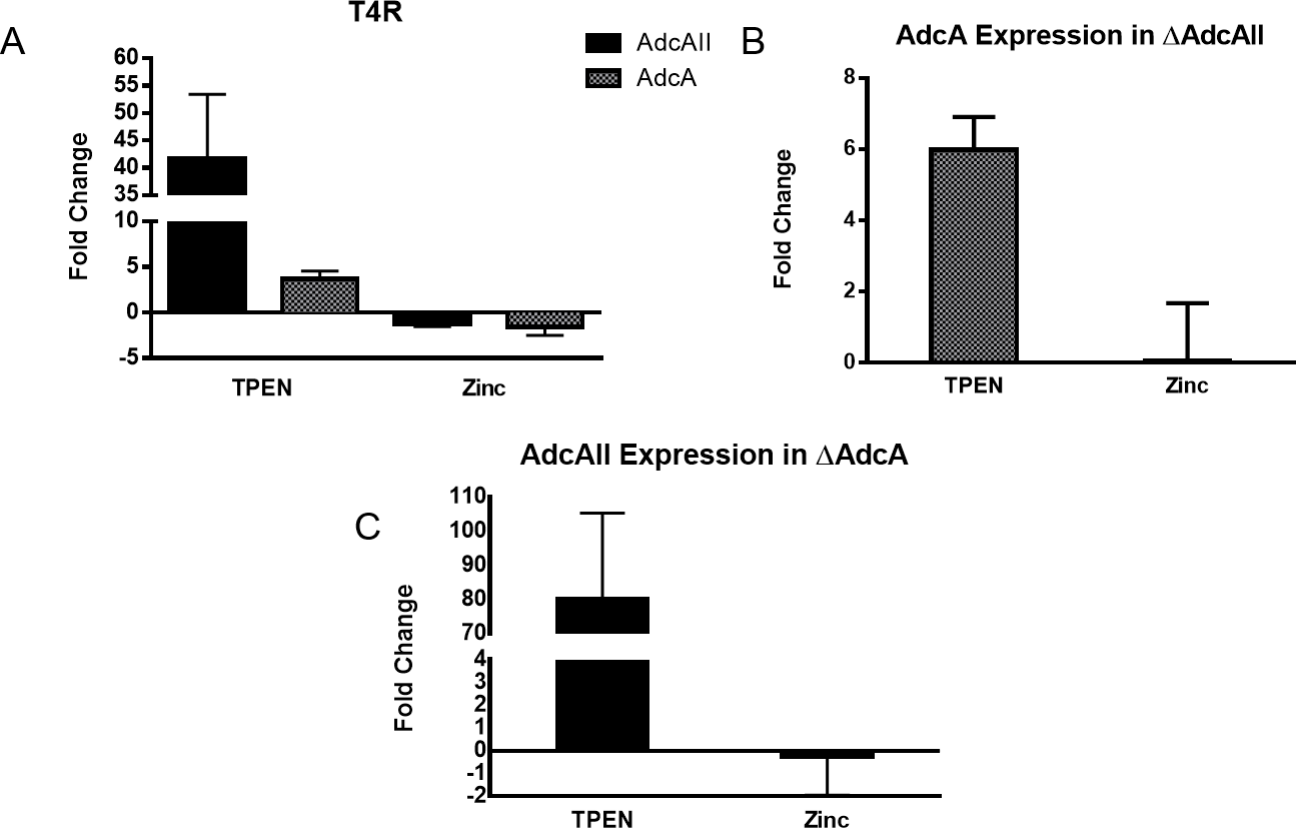
Effects of zinc on expression of *adcA* and *adcAII* in zinc rich (100μM ZnSO_4_) or low zinc environment (30μM TPEN). A) Expression of *adcA* (grey) and *adcAII* [28] in wild type T4R as measured by qRT-PCR ΔΔCT analysis. B) Expression of *adcA* in ΔAdcAII. C) Expression of a*dcAII* in ΔAdcA.

We also assessed the relative expression of *adcA* and *adcAII* under Zn^2+^-limiting and sufficient conditions. Addition of the Zn^2+^ chelator to wild type T4R resulted in 42-fold upregulation of *adcAII* but only 4-fold upregulation of *adcA* (Fig 2A). In the ΔAdcAII strain, *adcA* upregulation in the presence of the chelator was still only modest at 6-fold (Fig 2B); conversely, in the ΔAdcA strain, *adcAII* was strongly upregulated 80-fold in the presence of TPEN (Fig 2C). Thus, it appears that *adcAII* expression is more tightly coupled to Zn^2+^ concentration in the environment than *adcA*.

### Contribution of AdcA and AdcAII to colonization and invasion

**Fig 3:**
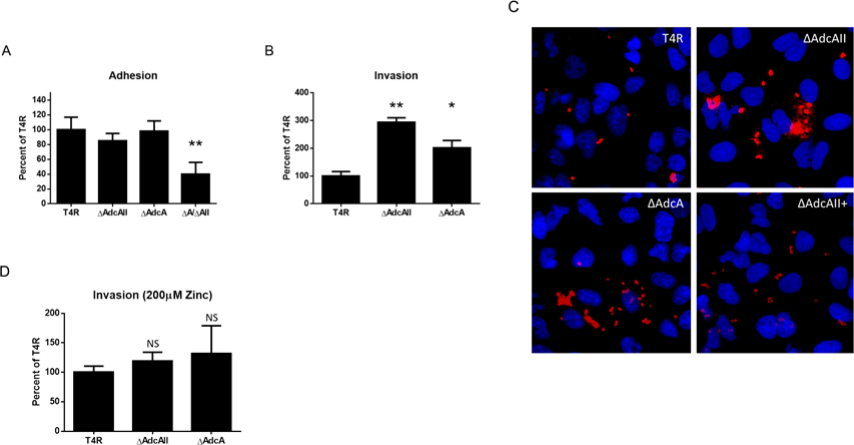
Effect of AdcA and AdcAII on adhesion and invasion in A549 cell line. A) Adhesion relative to T4R (100% representing approximately 0.9% of inoculum adhered). Bacteria were incubated with A549 cells for 30 mins in 24-well plates and adherent bacteria were quantitated. ** = P<0.0005. B) Invasion relative to T4R (100% representing approximately 0.03% of inoculum invaded). Bacteria were incubated for 2 hrs with A549 cells, followed by 1 hr in the presence of antibiotics to kill extracellular bacteria; cells were lysed, and intracellular bacteria were quantitated. * = P<0.005 and ** = P<0.0005. C) Fluorescent images of A549 cells incubated with T4R, ΔAdcA ΔAdcAII, ΔAdcAII+ as in B. Cells were washed, permeabilized, and bacteria were stained with anti-TEPC-15 antibody (in red). Cell nuclei are stained with DAPI (blue). D) Invasion relative to T4R (%). Bacteria were incubated for 2 hrs with A549 cells with 200μM ZnSO_4_, followed by 1 hr in the presence of antibiotics to kill extracellular bacteria; cells were lysed, and intracellular bacteria were quantitated. All experiments were conducted at least three times. Each experiment consisted of triplicate sample wells.

Homologs of AdcAII have been shown in related bacterial species to play a role in adhesion and invasion of host cells [6, 8, 29]. To determine if AdcAII serves a similar function for pneumococcus, we performed adhesion and invasion assays in the A549 human lung epithelial cell line. Interestingly, the loss of AdcAII led to a slight decrease in the number of bacterial cells adherent to A549 cells, while loss of AdcA led to no change in ability to adhere. Loss of both AdcA and AdcAII led to a significant decrease in adherence (Fig 3A; **p<0.0005). However, both ΔAdcA and ΔAdcAII strains were significantly more invasive in comparison to wild type T4R (Fig 3B; *p<0.005). The hyperinvasive phenotype of ΔAdcAII and ΔAdcA appears to be due to insufficient zinc acquisition. When zinc is supplemented at 200μM into media prior to invasion assays, the invasive phenotype of the mutants mimic that of the wild type (Fig 3D). Invasion data is not shown for the ΔAdcA/ΔAdcAII strain due to a growth defect of the strain during the two hr incubation required for invasion assays. We believe this to be a result of the mutant’s inability to acquire sufficient zinc since supplementation of 100μM ZnSO_4_ restored growth of the ΔAdcA/ΔAdcAII mutant (data not shown). Increased invasion of ΔAdcA and ΔAdcAII as compared to wild type T4R was also confirmed using fluorescent microscopy of A549 cells. The invasive phenotype of the complemented ΔAdcAII strain (ΔAdcAII+) returned closely to that of wild type T4R (Fig 3C). Western blot confirmed expression of AdcAII in our complemented ΔAdcAII+ strain, but expression was not equal to that of wild type T4R (S1 Fig). This could explain why invasion levels, though decreased, are not decreased to the same extent as that of wild type T4R.

Unencapsulated T4R from the TIGR4 strain background was used for these studies since encapsulated strains are known to poorly invade in vitro, and our goal was to specifically understand the role of surface proteins. Increased invasion was not due to growth differences between the strains as determined by comparing growth in F-12K (A549) media (data not shown).

**Fig 4:**
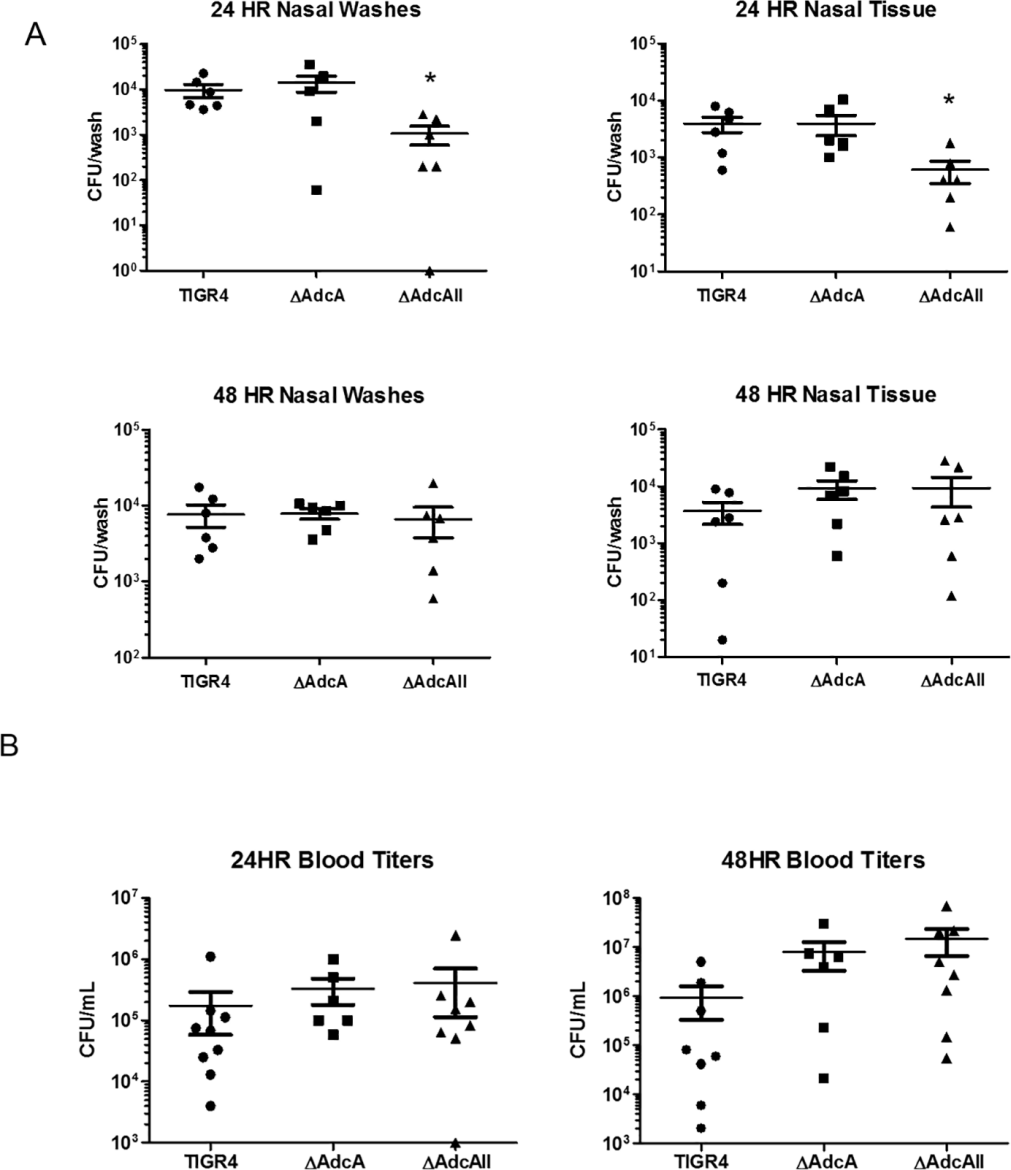
Effect of AdcAII on *in vivo* colonization and invasion. A) For colonization studies, mice were inoculated intranasally with bacteria (7x10^3^ CFU). At 24 and 48 hrs, mice were sacrificed and 200μL of PBS was passed from the trachea through the nose. Nasal passages were also collected, homogenized, and serial dilutions were plated. B) For invasion studies, mice were inoculated intranasally with bacteria (3.6x10^4^ CFU). Serial dilutions of blood samples were plated at 24 and 48 hrs. Results have been pooled from two independent experiments.

To assess the functions of AdcA and AdcAII *in vivo*, mice were challenged intranasally with *S*. *pneumoniae* TIGR4, T4ΔAdcA, and T4ΔAdcAII. Nasal washes, nasal tissues, and blood titers were collected at 24 and 48 hrs post-infection. The T4ΔAdcAII strain was found in significantly less abundance in nasal washes and nasal tissues taken 24 hrs post-inoculation; however, no significant differences were seen with T4ΔAdcA. However, at 48 hrs post-challenge, no significant differences were seen between TIGR4 and the mutants in either nasal washes or nasal tissues (Fig 4A). Thus, a modest colonization defect was seen only for T4ΔAdcAII and only early after challenge. Assessment of invasion as determined by blood bacterial counts demonstrated a trend for increased invasion for both T4ΔAdcA and T4ΔAdcAII, though these data did not reach statistical significance (Fig 4B). Similarly to the *in vitro* assays, the T4ΔAdcA/ΔAdcAII strain was not used during animal challenges due to growth deficiencies.

### Zinc-dependent gene expression

Microarray analysis was used to compare differential gene expression between TIGR4 and the ΔAdcAII strain. Ten genes were upregulated and twelve genes were downregulated in ΔAdcAII. Some of the genes upregulated in the ΔAdcAII strain include *phtD* and a v-type ATP synthase. Some of the genes that were the most strongly downregulated include neuraminidase B, a 50s ribosomal protein, and a sialidase B precursor (S2 Table). While the gene encoding *phtD* (SP1003) is directly downstream of the gene for *adcAII* (SP1002), upregulation was not due to polar effects since supplementation with zinc decreased the upregulation (S2 Fig). In order to investigate the role PhtD may have in the hyperinvasive phenotype of ΔAdcAII, we performed invasion assays as previously described. Interestingly, we saw that ΔPhtD had a similar phenotype to ΔAdcAII as it was approximately three times more invasive than wild type T4R (S3 Fig) (p<0.05). Thus indicating to us that though *phtD* is upregulated in ΔAdcAII, it is not responsible for the hyperinvasive phenotype.

**Fig 5:**
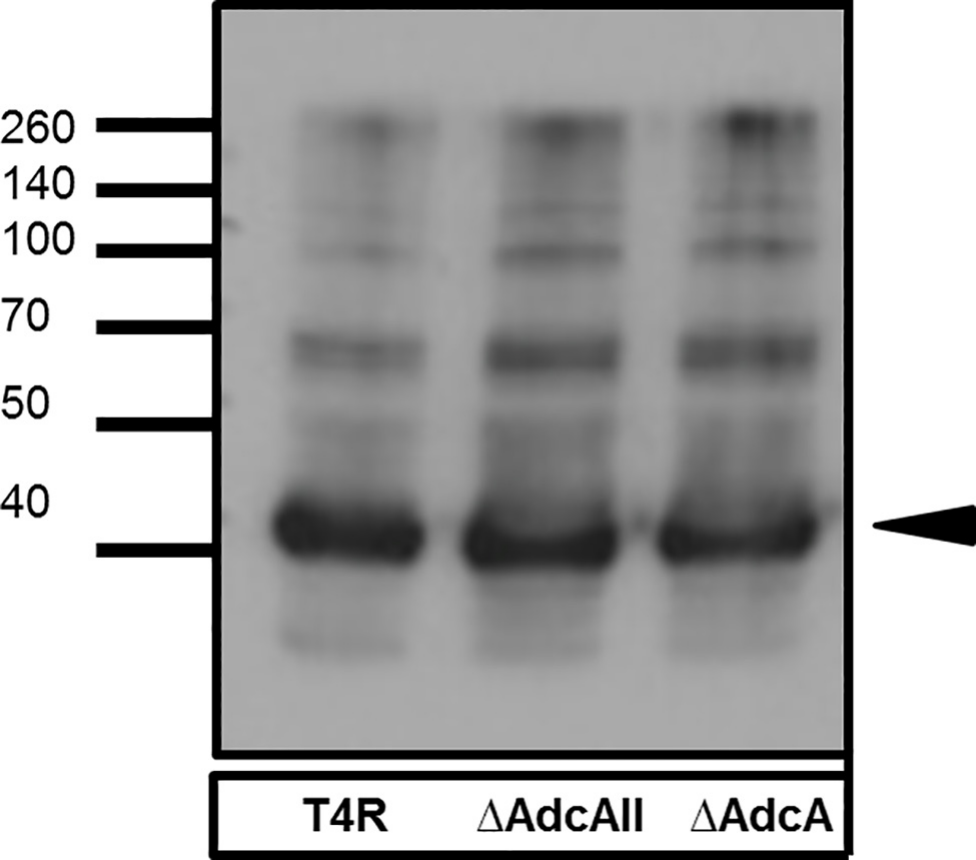
Western blot assessing pilus SP0463 expression. Lysates from T4R, ΔAdcA, and ΔAdcAII strains were blotted and probed with anti-SP0463 antibody and developed. Black arrow indicates SP0463 pilus monomer.

The two-component systems CbpR/S and HK/RR03 and the metalloregulators MerR and PsaR are known to down-regulate expression of pilus [30]. Mutations in these loci result in hyper-invasion of epithelial cells similar to that seen with ΔAdcAII [30]. This similarity suggested a potential role for AdcAII in regulating pilus gene expression. While microarray analysis did not identify changes in pilus gene expression in ΔAdcAII, western blot was used to assess expression of pilus in wild type T4R, ΔAdcA, and ΔAdcAII; however, no significant differences were seen (Fig 5).

### Assessment of potential laminin binding

**Fig 6:**
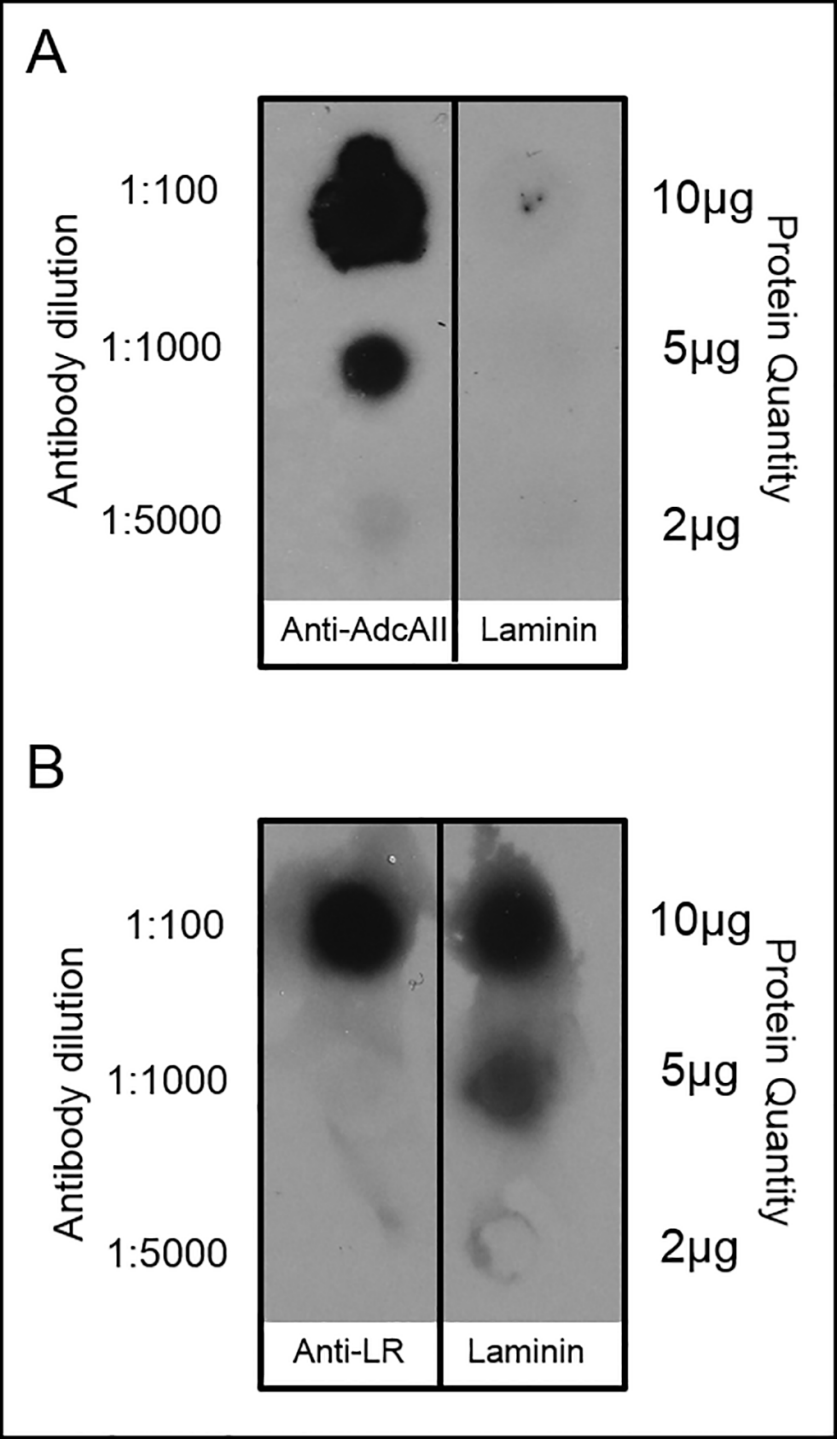
Dot blot demonstrating lack of laminin binding by AdcAII. A) Laminin (2 μg, 5 μg, and 10 μg spots) or anti-AdcAII antibody (1:100, 1:1000, 1:5000 spots) as control were probed with biotinylated recombinant AdcAII protein. B) To demonstrate laminin could interact with ligands using this method laminin protein (2 μg, 5 μg, and 10 μg spots) or anti-laminin receptor antibody (1:100, 1:1000, 1:5000 spots) as a control probed with recombinant laminin receptor and developed with anti-LR antibody.

AdcAII homologs, Lmb of *S*. *agalactiae* and Lbp or Lsp of *S*. *pyogenes*, have been shown to interact with the extracellular matrix protein laminin, increasing adhesion to host cells [5, 8, 29]. Given the degree of homology between AdcAII and these proteins (>60% identity), we investigated a potential role for AdcAII in interaction with extracellular matrix proteins. For these studies the *adcAII* gene from *S*. *pneumoniae* TIGR4 was expressed as a recombinant protein and purified by affinity chromatography. Using a dot blot technique, AdcAII failed to interact with human laminin (Fig 6) or the extracellular matrix proteins collagen or fibronectin (data not shown). Additionally, interaction of laminin and recombinant AdcAII by co-immunoprecipitation or ELISA could not be demonstrated (data not shown). Together, these data indicate that while highly homologous to laminin-binding proteins of related streptococci, AdcAII does not share an affinity for laminin.

### Biophysical Characterization of AdcAII

Circular dichroism (CD) spectroscopy and dynamic light scattering (DLS) were used to confirm the folded conformation of AdcAII. The far-UV CD spectrum of AdcAII suggests a folded structure (S4A Fig), and singular value decomposition (SVD) of CD data predict an α-helical content of 46% and a β-strand content of 7%. These values are in good agreement with the crystal structure of AdcAII (38% α-helix and 11% β-strand) [9]. The DLS results also suggest a compact, globular structure for purified AdcAII (S4B Fig). The observed hydrodynamic radius (R_H_) from DLS is 27.5 ± 0.4 Å, and this is also in good agreement with the radius predicted using the crystal structure (30.5 Å). These data strongly suggest that the protein in our studies is in its native confirmation; therefore, the lack of detectable binding to ECM components is not a result of misfolded or aggregated protein, but rather an intrinsic property of the protein itself.

## Discussion

The goal of the current study was to expand the possible functional roles of the *S*. *pneumoniae* Zn^2+^-binding lipoproteins AdcAII and AdcA from metal transport to virulence. Proteins homologous to AdcAII have been found to interact with the extracellular matrix protein laminin and contribute to virulence by participating in adhesion [5]. AdcA failed to show any significant phenotype in these activities and thus appears to be limited to metal transport. In contrast, AdcAII showed extensive roles in activities beyond metal transport. AdcAII did not associate with laminin as measured by affinity chromatography, dot blot analysis, and co-immunoprecipitation. However, loss of AdcAII attenuated adhesion and strongly increased invasion of host cells. This data combined with our data showing gene expression of *adcA*, *adcAII*, and *phtD* are zinc-dependent leads us to believe that the hyper-invasiveness of ΔAdcA and ΔAdcAII is a result of the zinc limitation. This is underlined by the fact that invasion assays demonstrate that the mutants are equally as invasive as wild type when zinc is supplemented to the media.

Over the course of progression from nasopharyngeal colonization to invasive disease the pneumococcus must respond to a broad range of Zn^2+^ concentrations [31]. The availability of Zn^2+^ is known to affect both colonization and development of invasive pneumococcal disease in mice [32, 33]. Zn^2+^ levels are generally thought to be higher in the nasopharynx and the lungs compared to levels found in the blood [34, 35]. Within the lungs, Zn^2+^ has been shown to be found at higher concentrations at the apical surface of the epithelium [36]. This suggests that the bacteria encounter higher levels of Zn^2+^ at the cellular surface prior to invasion into the blood stream [36]. Also, Plumptre *et al*. have shown that Zn^2+^ levels increase significantly in niches containing *S*. *pneumoniae* as early as 48 hrs post infection. Increased Zn^2+^ concentrations would decrease expression of the *adcA* and *adcAII* genes, and based on our results, may limit invasive potential of the bacteria. Though Plumptre *et al*. demonstrated this in the D39 background, our data support these findings in the TIGR4 background of *S*. *pneumoniae* as well [12].

Expression of AdcAII, the AdcCBA Zn^*2+*^ transporter operon, and all of the histidine triad proteins of pneumococcus are regulated by the Zn^2+^-dependent repressor AdcR [10, 17, 37]. This is consistent with AdcA and AdcAII serving as Zn^2+^ transporters and with their expression being reduced when Zn^2+^ levels are high. Bayle *et al*. have proposed that AdcA and AdcAII serve redundant roles in Zn^2+^ transport with one capable of substituting for the other when Zn^2+^ is low [11]. However, our results show a distinct effect of low Zn^2+^ on growth of mutants lacking only AdcAII. The differences between our findings and those of Plumptre et al, could be explained by differences in culture media between their group and ours. Alternatively, there could be AdcR-dependent differences in the strains R6 and T4R, though the sequences are nearly identical as determined through ClustalW alignment. Also, their studies identified that mutation of both AdcA and AdcAII resulted in complete loss of virulence in animal models of infection. This result is likely due to a complete inability of the bacteria to regulate intracellular Zn^2+^ concentrations *in vivo*. In contrast, our results with strains lacking AdcA or AdcAII alone demonstrate at least a trend for increased invasion both *in vitro* and *in vivo*. Plumptre *et al*. have performed challenge experiments with ΔAdcAII and ΔAdcA mutants in the D39 background and found similar results to ours with regards to colonization; however, their AdcAII mutant displayed a trend for decreased invasion at 24 hrs post-infection that then rebounded by 48 hrs to that of wild type[12]. In our model, the difference in invasion between ΔAdcAII and TIGR4 increased from 24 hrs to 48 hrs, indicating the relative importance of AdcA and AdcAII may be strain dependent.

In conclusion, we sought to characterize differences between AdcA and AdcAII in zinc homeostasis and contribution to virulence. This study has provided a link between expression of AdcAII, AdcA, and invasive potential of *S*. *pneumoniae*. It was determined that *S*. *pneumoniae* strains lacking either AdcAII or AdcA are more invasive than wild type strains. Additionally, AdcAII may be more important for both Zn^2+^ homeostasis and the regulation of pneumococcal virulence.

## Supporting Information

S1 Fig Western blot assessing AdcAII expression. Lysates from T4R, ΔAdcAII, and ΔAdcAII+ strains were blotted and probed with anti-LMB antibody and developed. Black arrow indicates AdcAII.
(TIF)

S2 Fig Effects of zinc on expression of *phtD*. A) Basal expression of *phtD* in wild type T4R vs ΔAdcAII as measured by qRT-PCR ΔΔCT analysis. B) Expression of *phtD* in wild type T4R vs ΔAdcAII when supplemented with 100μM ZnSO_4._
(TIF)

S3 Fig Effect of PhtD on invasion in A549 cell line. Invasion relative to T4R (%). Bacteria were incubated for 2 hrs with A549 cells, followed by 1 hr in the presence of antibiotics to kill extracellular bacteria; cells were lysed, and intracellular bacteria were quantitated. * = P<0.05. All experiments were conducted at least three times. Each experiment consisted of triplicate sample wells.
(TIF)

S4 Fig Biophysical characterization of AdcAII. A) Far-UV Circular Dichroism (CD) spectrum of 40 μM AdcAII at 25°C in 25mM Tris buffer pH 7.5 [28]. The reconstructed spectrum from singular-value decomposition (SVD) is also shown (red) to demonstrate good agreement between the observed data and secondary structure predictions. The data have been processed as described in materials and methods. B) Regularization fit results from dynamic light scattering (DLS) for AdcAII under identical conditions to (A). The average observed hydrodynamic radius (R_H_) is 27.5 ± 0.4 Å. This compares favorably with the crystal structure value of 30.5 Å.
(TIF)

S1 Table Mutagenesis Primers. (TIF)

S2 Table Microarray Analysis of TIGR4 & ΔAdcAII. Bacterial RNA was harvested at OD_600_ 0.5 and used to synthesize cDNA for hybridization to pneumococcal microarray.
(TIF)
