# Supplementary figures and images for: AdcAII of Streptococcus pneumoniae Affects Pneumococcal Invasiveness
Source: PLoS One. 2016 Jan 11;11(1):e0146785. doi: 10.1371/journal.pone.0146785 (PMC4709005; doi:10.1371/journal.pone.0146785)

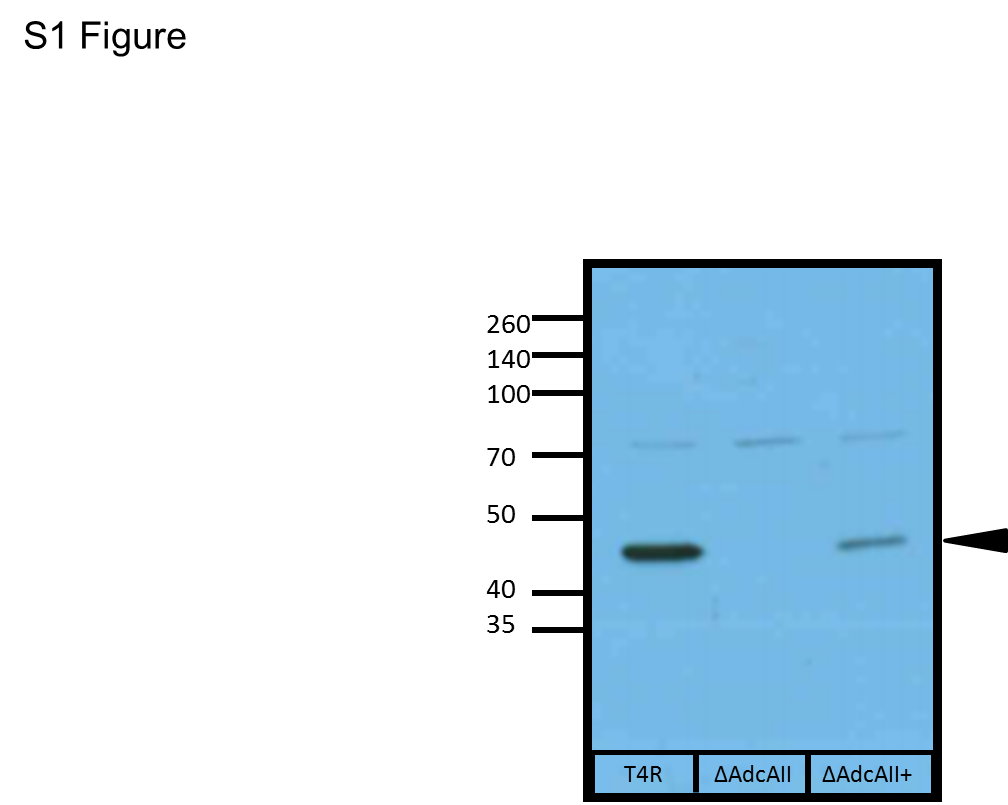

Supplement: S1 Fig — Lysates from T4R, ΔAdcAII, and ΔAdcAII+ strains were blotted and probed with anti-LMB antibody and developed. Black arrow indicates AdcAII. (TIF) [file pone.0146785.s001.tif]

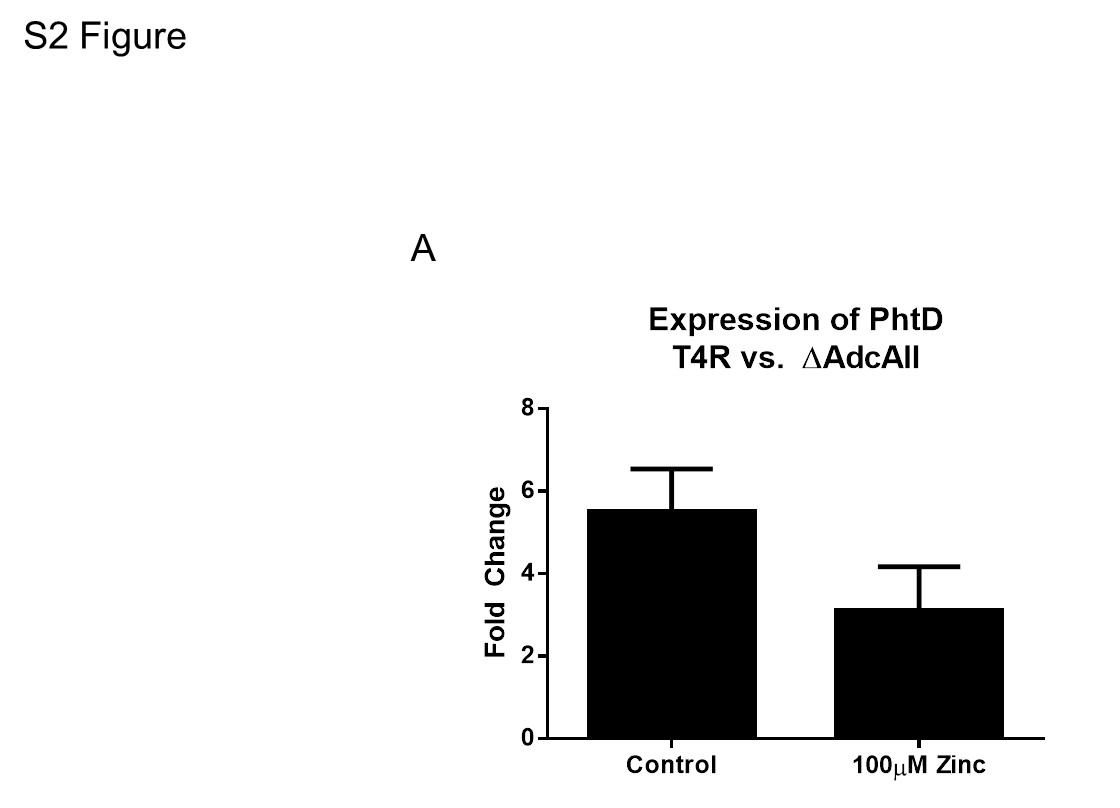

Supplement: S2 Fig — A) Basal expression of phtD in wild type T4R vs ΔAdcAII as measured by qRT-PCR ΔΔCT analysis. B) Expression of phtD in wild type T4R vs ΔAdcAII when supplemented with 100μM ZnSO4. (TIF) [file pone.0146785.s002.tif]

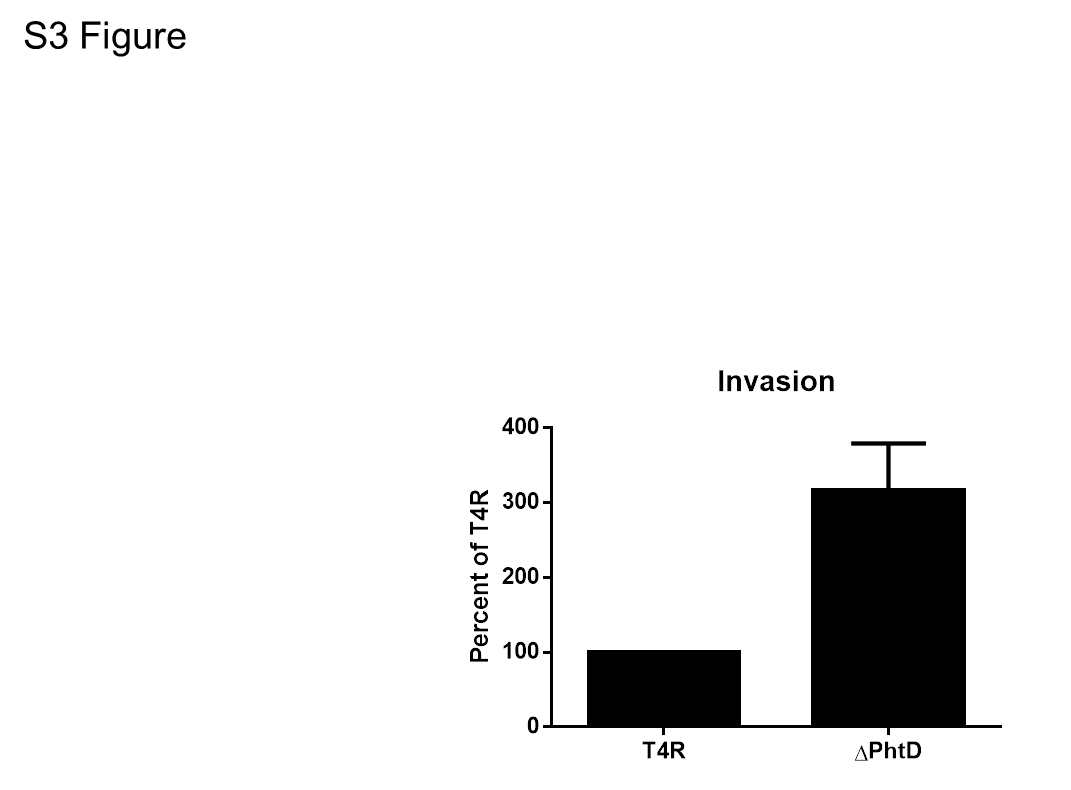

Supplement: S3 Fig — Invasion relative to T4R (%). Bacteria were incubated for 2 hrs with A549 cells, followed by 1 hr in the presence of antibiotics to kill extracellular bacteria; cells were lysed, and intracellular bacteria were quantitated. * = P<0.05. All experiments were conducted at least three times. Each experiment consisted of triplicate sample wells. (TIF) [file pone.0146785.s003.tif]

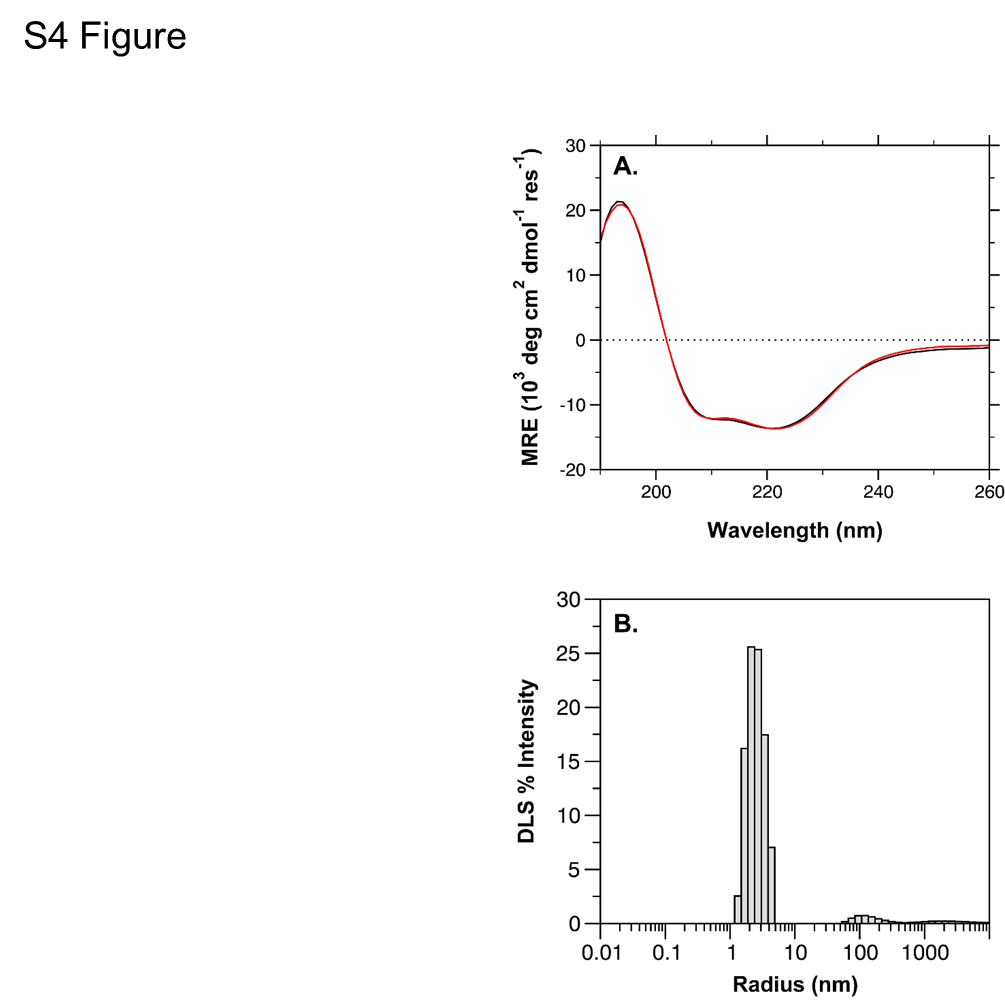

Supplement: S4 Fig — A) Far-UV Circular Dichroism (CD) spectrum of 40 μM AdcAII at 25°C in 25mM Tris buffer pH 7.5 [28]. The reconstructed spectrum from singular-value decomposition (SVD) is also shown (red) to demonstrate good agreement between the observed data and secondary structure predictions. The data have been processed as described in materials and methods. B) Regularization fit results from dynamic light scattering (DLS) for AdcAII under identical conditions to (A). The average observed hydrodynamic radius (RH) is 27.5 ± 0.4 Å. This compares favorably with the crystal structure value of 30.5 Å. (TIF) [file pone.0146785.s004.tif]

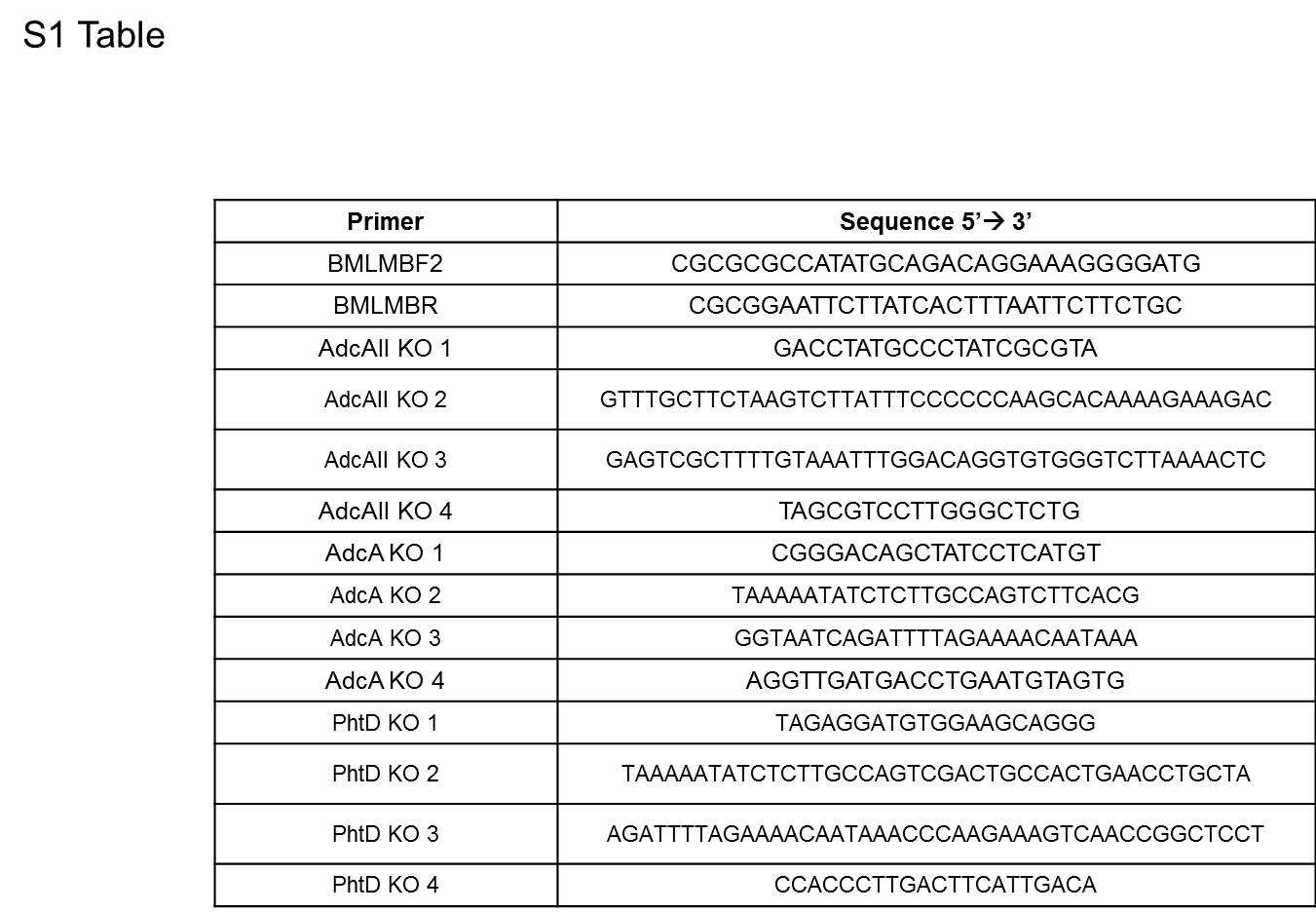

Supplement: S1 Table — (TIF) [file pone.0146785.s005.tif]

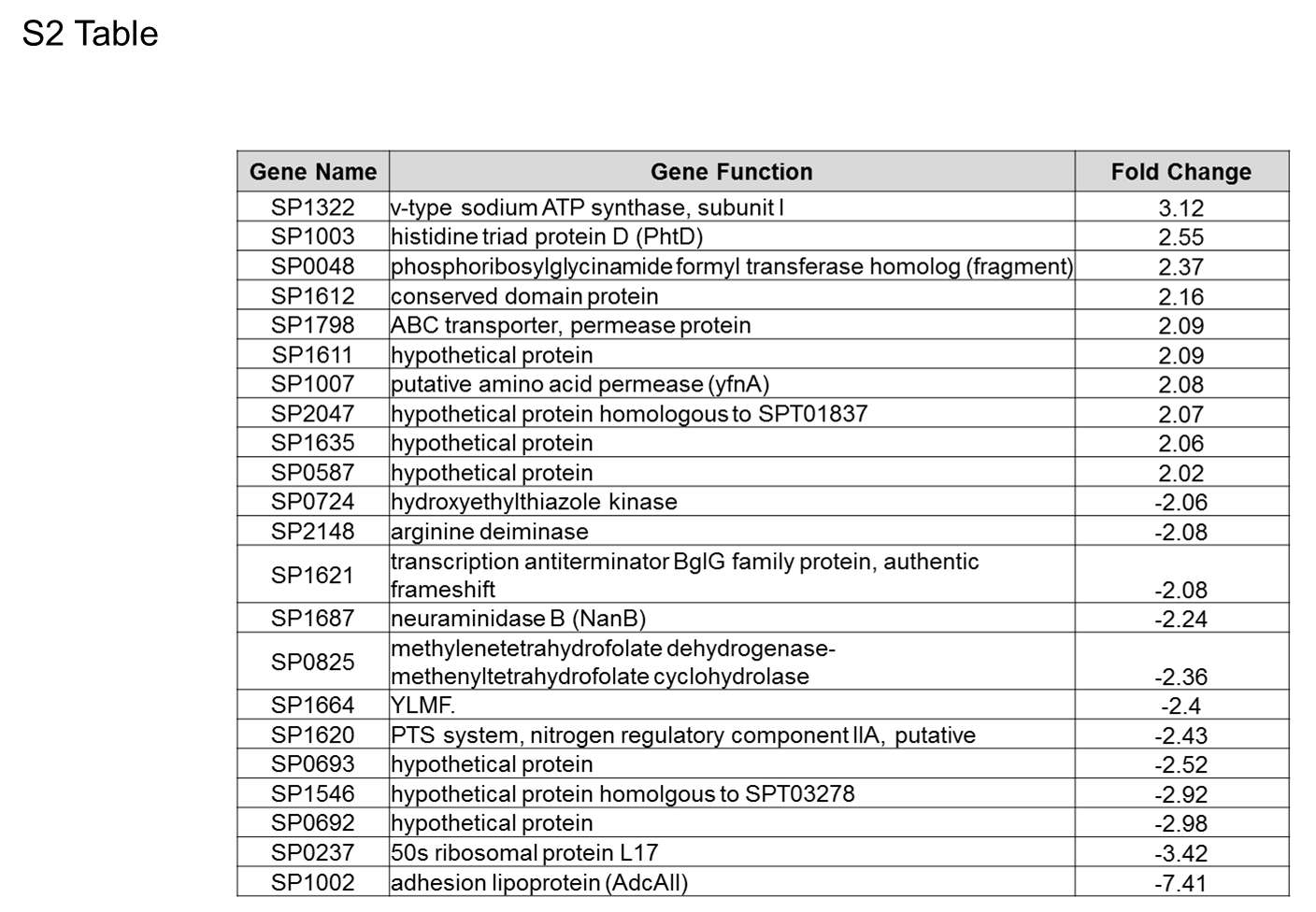

Supplement: S2 Table — Bacterial RNA was harvested at OD600 0.5 and used to synthesize cDNA for hybridization to pneumococcal microarray. (TIF) [file pone.0146785.s006.tif]
